# Screening for neonatal diabetes at day 5 of life using dried blood spot glucose measurement

**DOI:** 10.1007/s00125-017-4383-3

**Published:** 2017-08-05

**Authors:** Timothy J. McDonald, Rachel E. Besser, Mandy Perry, Tarig Babiker, Bridget A. Knight, Maggie H. Shepherd, Sian Ellard, Sarah E. Flanagan, Andrew T. Hattersley

**Affiliations:** 1 Blood Sciences, Template A2, Royal Devon and Exeter Hospital, Barrack Road, Exeter, EX2 5DW UK; 2 National Institute for Health Research (NIHR) Exeter Clinical Research Facility, University of Exeter, Exeter, UK; 3 Department of Paediatrics, University of Oxford, Oxford, UK; 4 Institute of Biomedical and Clinical Science, University of Exeter Medical School, Exeter, UK

**Keywords:** Blood spots, Glucose, Neonatal diabetes, Newborn, Screening

## Abstract

**Aims/hypothesis:**

The majority of infants with neonatal diabetes mellitus present with severe ketoacidosis at a median of 6 weeks. The treatment is very challenging and can result in severe neurological sequelae or death. The genetic defects that cause neonatal diabetes are present from birth. We aimed to assess if neonatal diabetes could be diagnosed earlier by measuring glucose in a dried blood spot collected on day 5 of life.

**Methods:**

In this retrospective case–control study we retrieved blood spot cards from 11 infants with genetically confirmed neonatal diabetes (median age of diagnosis 6 [range 2–112] days). For each case we also obtained one (*n* = 5) or two (*n* = 6) control blood spot cards collected on the same day. Glucose was measured on case and control blood spot cards. We established a normal range for random glucose at day 5 of life in 687 non-diabetic neonates.

**Results:**

All 11 neonates with diabetes had hyperglycaemia present on day 5 of life, with blood glucose levels ranging from 10.2 mmol/l to >30 mmol/l (normal range 3.2–6.0 mmol/l). In six of these neonates the diagnosis of diabetes was made after screening at day 5, with the latest diagnosis made at 16 weeks.

**Conclusions/interpretation:**

Neonatal diabetes can be detected on day 5 of life, preceding conventional diagnosis in most cases. Earlier diagnosis by systematic screening could lead to prompt genetic diagnosis and targeted treatment, thereby avoiding the most severe sequelae of hyperglycaemia in neonates.

## Introduction

Neonatal diabetes mellitus is a genetic disorder with an incidence of approximately 1 in 90,000 live births [1–3]. From birth there is severely reduced insulin production either due to reduced function or number of insulin producing beta cells. There are 23 known genetic causes that account for >80% of diabetes diagnoses in neonates <6 months of age [4]. Defining the genetic diagnosis is important as individuals with one of the two most common subtypes (*KCNJ11* and *ABCC8*) can achieve excellent glucose control with oral sulfonylurea tablets, avoiding a lifetime of insulin treatment and reducing the need for blood glucose monitoring [5–8].

At present, infants with neonatal diabetes are often unrecognised as being seriously ill until hyperglycaemia reaches life threatening levels. Typically, individuals present in the first 6 months (median age 6 weeks) with severe ketoacidosis associated with vomiting, dehydration, glucose >50 mmol/l and acidosis [8]. The treatment of paediatric diabetic ketoacidosis is extremely challenging; many babies die and those that do not may be left with life changing brain damage that requires lifelong institutional care [9–11]. These morbidity and mortality rates are likely to be a marked underestimate as brain damage and death from ketoacidosis in babies is often unrecognised or unrecorded [12].

The genetic defect causing neonatal diabetes is present within the developing pancreas in utero, leading to reduced insulin secretion from the fetal pancreas. Reduced insulin secretion may result in reduced insulin-mediated growth and hence a markedly lower than average birthweight in these individuals (2600 g compared with 3500 g in those unaffected) [13]. Insulin deficiency from birth will result in a high blood glucose level that could be detected in the first few days of life.

All newborns in the UK are offered a heel prick test where a small amount of blood is collected onto paper filter cards to test for nine rare but serious metabolic diseases [14]. The incidence of the conditions screened for range from 1:2000 to 1:300,000 live births [15]. Newborn screening presents a clear opportunity for systematic testing for the raised glucose seen in neonatal diabetes. Dried blood spots in the UK are collected from newborns at day 5–7 of life and sent to specialist screening laboratories by post. Upon receipt they are typically analysed the same day, as such a putative screening test would require the analyte to be stable at room temperature for at least 48 h.

The aim of this study is to establish the stability of blood spot glucose and to assess the diagnostic accuracy of blood spot glucose collected at day 5 of life for detecting neonatal diabetes.

## Methods

### Participants

#### Glucose stability study

Twenty volunteers were recruited for the glucose stability study as part of the Exeter 10,000 Project undertaken at the Exeter NIHR Clinical Research Facility (Exeter, UK). Volunteers were identified by research nurses and residual blood samples were used to make blood spots.

The study was approved by the Frenchay South West National Research Ethics Service Committee, UK (REC 09/H0106/75). Written informed consent was obtained from all participants.

#### Establishing a glucose normal range for neonates at day 5 of life

Families were recruited as part of the Exeter Family Study of Childhood Health (EFSOCH), a 5 year prospective study started in 1999, with the aim of examining genetic influences on fetal and early growth [16]. As part of this study all newborns (*n* = 687) had heel prick capillary blood measured at day 5 of life at the same time that a dried blood spot card was collected.

#### Recruitment of infants with neonatal diabetes

From a cohort of 170 infants in the UK with genetically confirmed neonatal diabetes we contacted the referring clinicians of 42 of the most recently diagnosed (diagnosed between 2009 and 2015) to request permission to retrieve the dried blood spot cards for blood spot glucose analysis. If permission was received, the relevant screening laboratory was contacted and one of the participant’s original dried blood spots, and also one/two anonymous control blood spots from infants who had their dried blood spot cards collected on the same day, were sent to Exeter for glucose analysis.

### Materials

Analyses were undertaken in the Blood Sciences Research Laboratory of Royal Devon and Exeter Hospital (Exeter, UK) or the Derriford Combined Laboratories (Plymouth, UK).

#### Blood spot elution

Blood spot eluent was prepared by punching a 3 mm blood spot from each participant’s dried blood spot card, to which 125 μl of a 2% trichloroacetic acid solution (Sigma-Aldrich, Dorset, UK) was added and left at room temperature for 45 min. Samples were subsequently centrifuged at 1500 *g* for 1 min and the supernatant fraction was transferred for glucose analysis.

#### Glucose analysis

Blood spot eluent glucose levels were measured using a manual rate-reaction hexokinase method (Randox, Belfast, UK) on a PerkinElmer Lambda 20 UV/VIS Spectrometer (PerkinElmer, London, UK). Measured eluent glucose levels were related back to capillary glucose levels using a one point calibration (calibration blood spot prepared using a 50% hematocrit-adjusted glucose calibration material [Randox]). The total intra-assay CV for blood spot glucose (including extraction) is 10.3% at 3 mmol/l and 15% at 14 mmol/l.

### Methods

#### Establishing the stability of glucose on dried blood spots

From each participant (*n* = 20) we generated 16 blood spots (approximately 1.5 ml of blood) using standard dried blood spot cards. At the time of collection the first blood spot was allowed to dry for 2 h and then eluted and analysed for baseline glucose. The remaining 15 blood spots from each participant were split into three groups, with one-third (*n* = 5) stored at room temperature, one-third (*n* = 5) in the fridge (4°C) and one-third (*n* = 5) in the freezer (−20°C). One blood spot from each temperature condition was then analysed for glucose at 1, 2, 3, 7 and 14 days post initial collection to establish a stability profile.

#### Establishing a glucose normal range for neonates at day 5 of life

As part of the EFSOCH study [16] each newborn (*n* = 687) had heel prick capillary blood glucose measured on the Bayer Elite glucose meter (Bayer, Newbury, UK) at day 5 of life [17].

#### Dried blood spot glucose levels in infants with neonatal diabetes

Dried blood spots from UK infants with neonatal diabetes and paired control samples had been stored for variable lengths of time (see Table 1) and under variable conditions (ambient temperature and refrigerated) leading to variable glucose degradation. To allow for this variable degradation, we adjusted blood spot glucose for each participant to account for the fall in the glucose values seen in the simultaneous control samples. Therefore, for each participant an adjustment factor was calculated using data from the simultaneous control samples:

1. Adjustment factor = mean glucose of reference range (4.6 mmol/l) / mean glucose of all simultaneous control samples
2. Adjusted glucose = measured blood spot glucose × adjustment factor.

**Table 1:** Demographics of infants with neonatal diabetes assessed for dried blood spot glucose at day 5

Gene	Sex	Current age (years)	Status	Age at diagnosis (days)	Birthweight (g)	Gestation (weeks)	Projected blood spot glucose (mmol/l)
*6q24*	Female	3	TNDM	2	1353	36	23.3
*6q24*	Female	2	TNDM	2	2095	36	12.6
*6q24*	Male	1	TNDM	12	1660	35	28.6
*6q24*	Male	3	TNDM	21	1990	40	>30
*ABCC8*	Male	7	TNDM	56	2500	40	27.4
*GATA6*	Female	3	PNDM	6	1200	37	12.6
*GCK*	Female	5	PNDM	112	2080	42	18.7
*GCK*	Female	2	PNDM	2	1680	38	17.1
*GLIS3*	Female	5	PNDM	1	1170	35	10.2
*GLIS3*	Female	1	PNDM	2	1860	39	10.6
*KCNJ11*	Female	5	PNDM	56	2270	41	>30

PNDM, permanent neonatal diabetes mellitus; TNDM, transient neonatal diabetes mellitus

This analysis made the following assumptions: (1) degradation of blood spot glucose was proportional in case and control samples, (2) control blood spot glucose reflected the mean glucose of the normal range for neonates at day 5 of life.

### Statistical analyses

For the stability study, glucose results are presented as mean percentage change from baseline with 95% CI. Percentage changes of glucose >10% from baseline concentrations were considered clinically significant. Differences between the baseline and the level of glucose at 14 days were assessed by the Wilcoxon test.

A normal range was established for newborns at day 5 of life by calculating the mean and the range incorporated by 1.96 SD each side of the mean. Comparison of glucose level on blood spots from infants with and without neonatal diabetes is presented as medians and range, and differences between these two groups were assessed using Mann–Whitney *U* test.

## Results

### Two week stability experiment

**Fig. 1:**
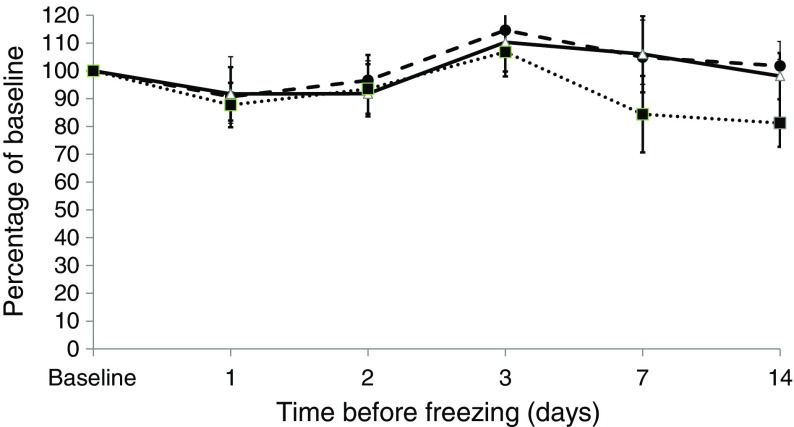
Stability of blood spot glucose stored at room temperature (dotted black line/squares), in a fridge (4°C, solid black line/triangles) and in a freezer (−20°C, dashed line/circles) over 14 days. Blood taken from 20 volunteers. Data presented as mean percentage of baseline at each time point, with error bars representing the 95% CI

The stability of blood spot glucose under three storage conditions (room temperature, fridge [−4°C] and freezer [−20°C]) over 14 days is shown in Fig. 1. Under all conditions samples were stable for over 3 days. When stored in the fridge or freezer there was no significant degradation over 14 days (98% of baseline CI 84, 112 and 101% of baseline CI 88, 115, respectively). At room temperature samples had degraded to 84% (*p* = 0.05) at 7 days and 81% (*p* = 0.001) at 14 days.

### Capillary blood glucose normal range for neonates at day 5 of life

The mean glucose measured in 687 neonates at day 5 of life was 4.6 mmol/l with an SD of 0.7 mmol/l. This gives a normal range calculated by the mean ± 1.96 SD of 3.2–6.0 mmol/l. The highest glucose measured in the group was 7.0 mmol/l.

### Blood spot glucose levels in infants with neonatal diabetes

Dried blood spots from 11 infants with confirmed monogenic neonatal diabetes were received (see Table 1 for participant characteristics). All 11 participants had intra-uterine growth retardation and birthweights below the second centile for gestation and sex. Age at diagnosis ranged from 2 days to 112 days after birth.

Participants with six different genetic aetiologies were assessed, including two with a *KCNJ11* or *ABCC8* mutation, which account for >40% of all reported neonatal diabetes diagnoses.

**Fig. 2:**
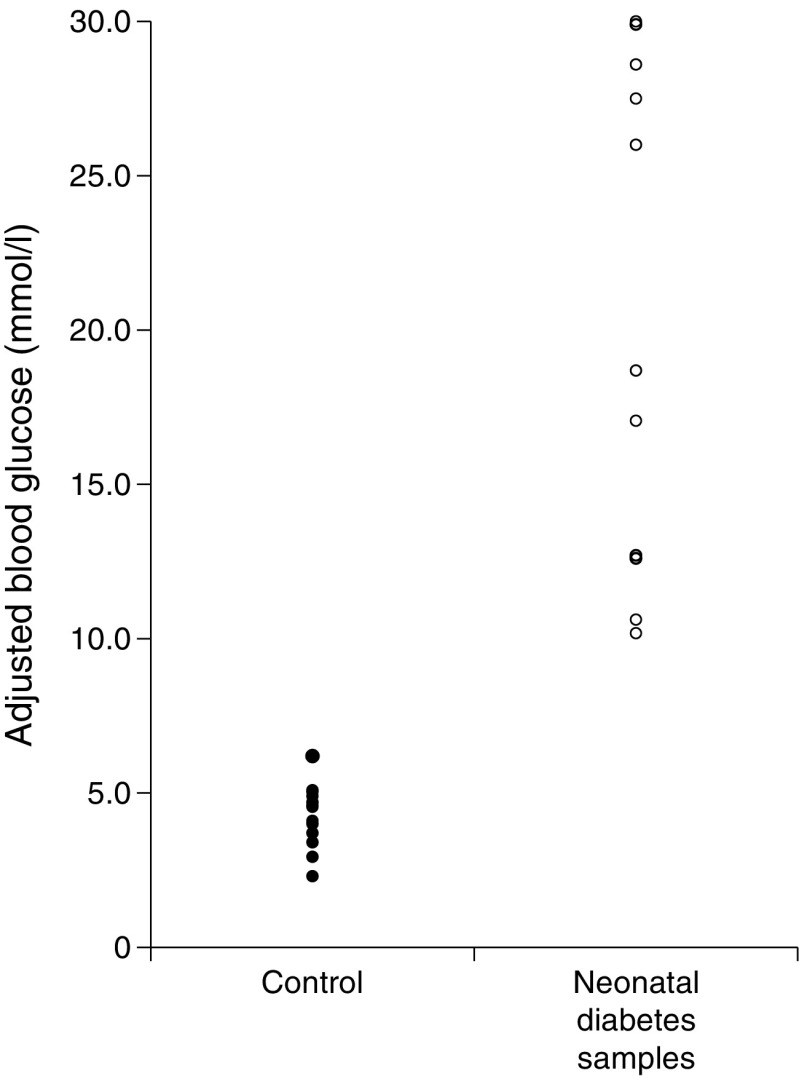
Graph showing projected glucose (adjusted for degradation with time) in Guthrie blood spots collected at day 5 of life for participants with neonatal diabetes and paired control samples collected on the same day

With each neonatal diabetes blood spot we received one (*n* = 5) or two (*n* = 6) control Guthrie blood spot samples collected on the same day. All neonatal diabetes case samples had higher glucose than the matched control samples at day 5 of life (see Fig. 2). The median projected glucose of the paired control samples was 4.6 mmol/l (2.3–6.2 mmol/l) compared with 18.7 mmol/l (10.2– >30.0 mmol/l) for the neonatal diabetes case samples (*p* < 0.001). All the infants with neonatal diabetes had glucose levels that were above the normal range of 3.2–6.0 mmol/l, with the lowest being 6 SD above the mean of the normal range (10.2 mmol/l). The difference between the highest blood spot glucose level of all 687 control samples used in the normal range and the lowest glucose level for an infant with neonatal diabetes was 3.2 mmol/l or 4.6 SD. There was no association between glucose level and age of diagnosis for the infants with neonatal diabetes (*p* = 0.47).

## Discussion

We have shown that blood glucose in infants with a genetic diagnosis of neonatal diabetes is raised at day 5 of life and can be detected using blood spot glucose collected as part of the UK newborn screening programme.

In total we assessed the newborn screening dried blood spots from 11 infants with neonatal diabetes. All had significantly raised blood glucose at day 5 of life and in six participants this preceded the clinical diagnosis by up to 16 weeks. A study of 1030 infants with neonatal diabetes showed that the mean age of diagnosis for neonatal diabetes is 6 weeks. Of these infants, 79% were diagnosed after day 5 of life [4]. This suggests that newborn screening will be able to identify neonatal diabetes in the majority of infants before they develop overt diabetes. Early diagnosis of hyperglycaemia would allow rapid genetic testing and early implementation of treatment, potentially avoiding the severe morbidity and mortality rates associated with the late presentation of diabetes.

A screening strategy requires the test analyte to be stable for 48 h. Dried blood spots in the UK are collected from newborns at day 5–7 of life and sent to specialist screening laboratories using the postal service. Upon receipt blood spots are typically analysed the same day with abnormal results called through to healthcare teams immediately. As such a putative screening test would require the analyte to be stable at room temperature for at least 48 h. We found that blood spot glucose is stable in all conditions for 3 days. At room temperature there is a 16% degradation after 7 days. The 3 day stability of blood spot glucose makes it a feasible test that could practically be measured within the time restrictions of the current UK newborn screening programme.

Screening for the early hyperglycaemia in neonatal diabetes could theoretically be achieved with a point of care testing (POCT) blood glucose or even a local laboratory glucose. This has the benefit of reducing the time it takes to send the sample to the screening laboratory and receiving the abnormal result. However, in practice this would be expensive to implement and very difficult to monitor and ensure all babies have been tested systematically with the appropriate follow-up clinical care pathway for abnormal results. The benefit of adding glucose to the additional newborn screening test repertoire is that all the infrastructure for sample collection, quality control and clinical reporting are in place and robustly monitored.

This study provides preliminary data that suggests it may be possible to systematically screen for neonatal diabetes. We found that all infants with neonatal diabetes in our cohort had blood spot glucose >3 SD above the highest glucose in the control cohort. This suggests that systematic blood spot glucose screening would have a high diagnostic accuracy for identifying infants with undiagnosed neonatal diabetes. A screening strategy would also ensure systematic genetic testing, either on the dried blood spot or a repeat blood sample, to ensure the correct genetic aetiology is established and, consequently, the most effective treatment is implemented, such as sulfonylurea therapy in individuals with *ABCC8* or *KCNJ11* mutations which can also have neurological as well as glycaemic therapeutic effect. Therefore, systematic screening could result in not only reduced mortality and morbidity rates but also considerable savings through avoiding lifetime disability and premature death.

When considering the implementation of a new screening test, it is not enough to diagnose the condition alone. There is a requirement to show that making the diagnosis will prevent avoidable complications and death in an acceptable and cost-effective manner. For a specific test to be considered viable for a screening programme, a number of criteria have been suggested. The most widely used are those proposed by Wilson–Jungner, which are designed to appraise the validity of a screening programme [18] and have been adapted for application to screening in the UK [19]. The original criteria are:

1. the condition being screened for should be an important health problem;
2. the natural history of the condition should be well understood;
3. there should be a detectable early stage;
4. treatment at an early stage should be of more benefit than at a later stage;
5. a suitable test should be devised for the early stage;
6. the test should be acceptable;
7. intervals for repeating the test should be determined;
8. adequate health service provision should be made for the extra clinical workload resulting from screening;
9. the risks, both physical and psychological, should be less than the benefits;
10. the costs should be balanced against the benefits.

Screening for neonatal diabetes clearly fulfils criteria 1–4, with compelling evidence that early identification of neonatal diabetes would avoid many of the severe complications and deaths associated with unrecognised early presentation of diabetes. The results of this work provide preliminary evidence that a suitable, acceptable and low-risk test exists that can identify neonatal diabetes in a newborn screening programme.

This study has limitations. We assessed the blood spot glucose in a relatively small number of infants with neonatal diabetes. To confirm the diagnostic accuracy of a screening strategy, a larger numbers of samples will need to be assessed. Ideally this will occur close to diagnosis to reduce the degradation seen in storage. The tests were performed retrospectively and the glucose adjusted to the mean control values were the mean of a population of 5-day-old infants, this required us to make a number of assumptions on the linear degradation of blood spot glucose and the mean blood spot glucose in the pair control samples.

A large prospective assessment of the screening strategy will be required to confirm that blood spot glucose is a viable screening strategy for neonatal diabetes and this will need to be accompanied by a health economic analysis to show cost-effectiveness and take into account that some infants with neonatal diabetes are diagnosed before day 5 of life [20]. In addition, not all the newborn screening programmes around the world are collected on day 5, with some collected as early as day 2–3, as such this needs to be modelled into the screening performance when undertaking a prospective study and economic evaluation. Finally, the normal range for blood glucose in the 687 infants at day 5 were analysed on capillary blood and whilst this demonstrates tight glycaemic control in this age group, this is a different methodology from dried blood spot glucose and therefore direct comparisons may not be appropriate and a normal range on dried blood spot needs to be derived.

In conclusion, neonatal diabetes can be detected on day 5 of life by glucose testing on dried blood spot cards. This is preliminary evidence to support further consideration of newborn screening. If glucose testing was performed routinely at 5 days, it could result in infants being diagnosed before they present with symptoms from diabetic ketoacidosis and hence avoid the permanent and long-term neurological sequelae.

## Abbreviation

EFSOCH: Exeter Family Study of Childhood Health
